# Public interest trends for COVID-19 and pandemic trajectory: A time-series analysis of US state-level data

**DOI:** 10.1371/journal.pdig.0000462

**Published:** 2024-03-12

**Authors:** Panayiotis D. Ziakas, Eleftherios Mylonakis

**Affiliations:** 1 Department of Medicine, Brown University, Providence, Rhode Island, United States of America; 2 Department of Medicine, Houston Methodist Hospital, Houston, Texas, United States of America; Tsinghua University, CHINA

## Abstract

*Google Trends* provides spatiotemporal data for user-specific terms scaled from less than 1 (lowest relative popularity) to 100 (highest relative popularity) as a proxy for the public interest. Here we use US state-level data for COVID-19 to examine popularity trends during the pandemic evolution. We used "coronavirus" and "covid" search terms and set the period up from January 1st, 2020, to November 12, 2022. We measured the agreement on web rankings between states using the nonparametric Kendall’s W (0 for no concordance to 1 for perfect agreement). We compiled state-level weekly data on COVID-19 incidence and mortality and scaled state curves from 0 to 100 through a min-max normalization process. We used a dynamic time-warping algorithm to calculate similarities between the popularity, mortality, and incidence of COVID-19. The methodology is a pattern recognition process between time series by distance optimization. The similarity was mapped from 0 to 1, with 1 indicating perfect similarity and 0 indicating no similarity. The peak in popularity was in March 2020, succeeded by a decline and a prolonged period of fluctuation around 20%. Public interest rose briefly at the end of 2021, to fall to a low activity of around 10%. This pattern was remarkably consistent across states (Kendal’s W 0.94, p < 0.001). Web search trends were an impression of contagion growth: Overall, popularity-mortality trajectories yielded higher similarity indices (median 0.78; interquartile range 0.75–0.82) compared to popularity-incidence trajectories (median 0.74; interquartile range 0.72–0.76, Wilcoxon’s exact p<0.001). The popularity-mortality trajectories had a very strong similarity (>0.80) in 19/51 (37%) regions, as opposed to only 4/51 (8%) for popularity-incidence trajectories. State-level data show a fading public concern about COVID-19, and web-search popularity patterns may reflect the COVID-19 trajectory in terms of cases and mortality.

## Author summary

We analyzed public interest for COVID-19 across 50 states and the District of Columbia through web search query data. We found an analogous longitudinal pattern across states, characterized by a maximum interest during the initial epidemic waves that attenuated during the subsequent waves and declined to a minimum by the end of 2022. Through a distance optimization process, we showed that relative web search popularity was a good proxy for contagion growth, namely the mortality and incidence trajectories. Overall, the popularity-mortality trajectories had higher similarity compared with popularity-incidence trajectories. Web search data could capture the pandemic growth aside from standard epidemiological measures.

## Introduction

Mining search engine data has provided insights into important health issues, such as the epidemiology of cancer [1], infectious diseases [2], vaccination campaigns [3], or monitoring interest in prescribed drugs [4]. Specifically, web search engines and social media activity can serve as early warning signals for infectious disease outbreaks, case forecasting, and hot-spot identification and complement traditional surveillance methods. Google Trends data have been used for correlation and forecasting studies in influenza outbreaks [5], Zika virus [6], Middle East respiratory syndrome (MERS) [7], measles [8], and, recently, COVID-19 [9]. Diverse analytic methodologies, language restrictions, and testing policies preclude a unified evaluation. However, the findings were supportive across various geographic regions, and the majority of studies (83%) incorporating web search data concur on the applicability of search engines when studying the COVID-19 pandemic [9]. Despite the limitations associated with big data [10,11], in situations of profound population concern, such as the COVID-19 pandemic, examining online browsing behavior may help understand disease trajectory.

We recently showed that at the national level, web search popularity patterns for COVID-19 were similar across major Western countries and the United States [12]. These patterns reflected the pandemic course, and preferentially aligned with the mortality trajectory rather than the incidence trajectory [12]. The findings provide the rationale to explore if these associations would also stand valid at the subnational level, namely across smaller geographic divisions. *Google* (Google Inc., Mountain View, CA, USA) is the most popular platform for surveying public behavior and attracts the majority of web searches [13]. We used *Google Trends* to monitor state-level public interest in COVID-19 in the United States. We aimed to measure the similarity of web search behaviors across states and the potential link to the course of COVID-19, including incidence and mortality.

## Method

### State-level public interest for COVID-19

The *Google Trends* tool provides a longitudinal profile of the relative popularity of a given search query, scaled from <1 (lowest) to 100 (highest) for the user-defined period. We used "coronavirus" and "covid" combined as search query (coronavirus + covid). The *Google Trends* tool accepts combinations of search terms in a single query using the “+” sign which acts as an OR Boolean operator, to produce a single, normalized time-series of public interest. The analysis period covered January 1, 2020, to November 12, 2022, in the United States. We retrieved the pertinent outputs to create a state-level time series of relative popularity. A search query generates the relative popularity report from a random sample of an anonymous database, corresponding to the geographic and temporal selection. As sampling bias could influence subsequent searches, we obtained multiple datasets after cache refresh to validate results, using the same query and time limits. The first state-level output served for primary analysis (index dataset), and the remaining datasets for validation. We used nonparametric Kendall’s W to test the concordance of rankings between states regarding web search popularity. The test statistic ranges from 0 to 1, with 1 indicating perfect agreement and 0 indicating perfect disagreement, and no prior knowledge about the data distribution is required [14].

### Comparison between public interest and Covid-19 trajectory across states

In addition, state-level time series data on COVID-19 reported incident cases and deaths up to October 22, 2022 were extracted from the CDC website [15]. We compared the relative popularity of web searches to the course of COVID-19, namely weekly mortality and incident cases. We initially converted the daily time series on cases and deaths to weekly estimates and then transformed (scaled) the output to a range between 0 to 100 using min-max normalization. The curated data allow a direct comparison of the web search popularity with mortality and incidence trajectories, and conserve the original shape and outlier influence [16]. The similarity spans 0 to 1, with 1 indicating perfect similarity and 0 indicating no similarity. A similarity index >0.80 was considered a very good agreement.

We used the Canberra distance between time series to evaluate similarity, given that it maps within the bounds of 0 to 1 and performs better with non-negative numbers and rankings [17,18]. We selected a dynamic time-warping algorithm for optimal placement of time series that allows pattern recognition and is robust against outliers, shifts, or transformations [19]. We set warping time up to 8 weeks as a more conservative scenario for time-series alignment, to avoid unconstrained alignment beyond epidemiologically irrelevant lags. The extended methodological framework is available in our recent publication on national-level data [12]. The maximum warping time was optimized through a simulation process, taking into account the lag between case confirmation and deaths [12]. Of note, we used the same search terms and warping parameters in the present study to secure consistency and validity of interpretation between studies. We calculated the similarity by subtracting the mean distance between time series from unity. The ’dtw package’ [20] in R served for data analysis [21]. All raw data were open-source, open-access, anonymized registry data, free to use, process, and distribute.

## Results

There was near-perfect concordance of web search popularity for COVID-19 across states (Kendal’s W 0.94, p<0.001). We ran 15 identical queries after cache refresh between November 22, 2022 and February 4, 2023 and concordance did not alter at a sensitivity level of 10^−3^, indicating lack of sampling bias. Estimates did not vary after sequentially excluding single-state data and repeating the process in the index dataset, which argues against the presence of influential observations and outliers (all changes are limited again to the third decimal point, from 0.935–0.937).

**Fig 1:**
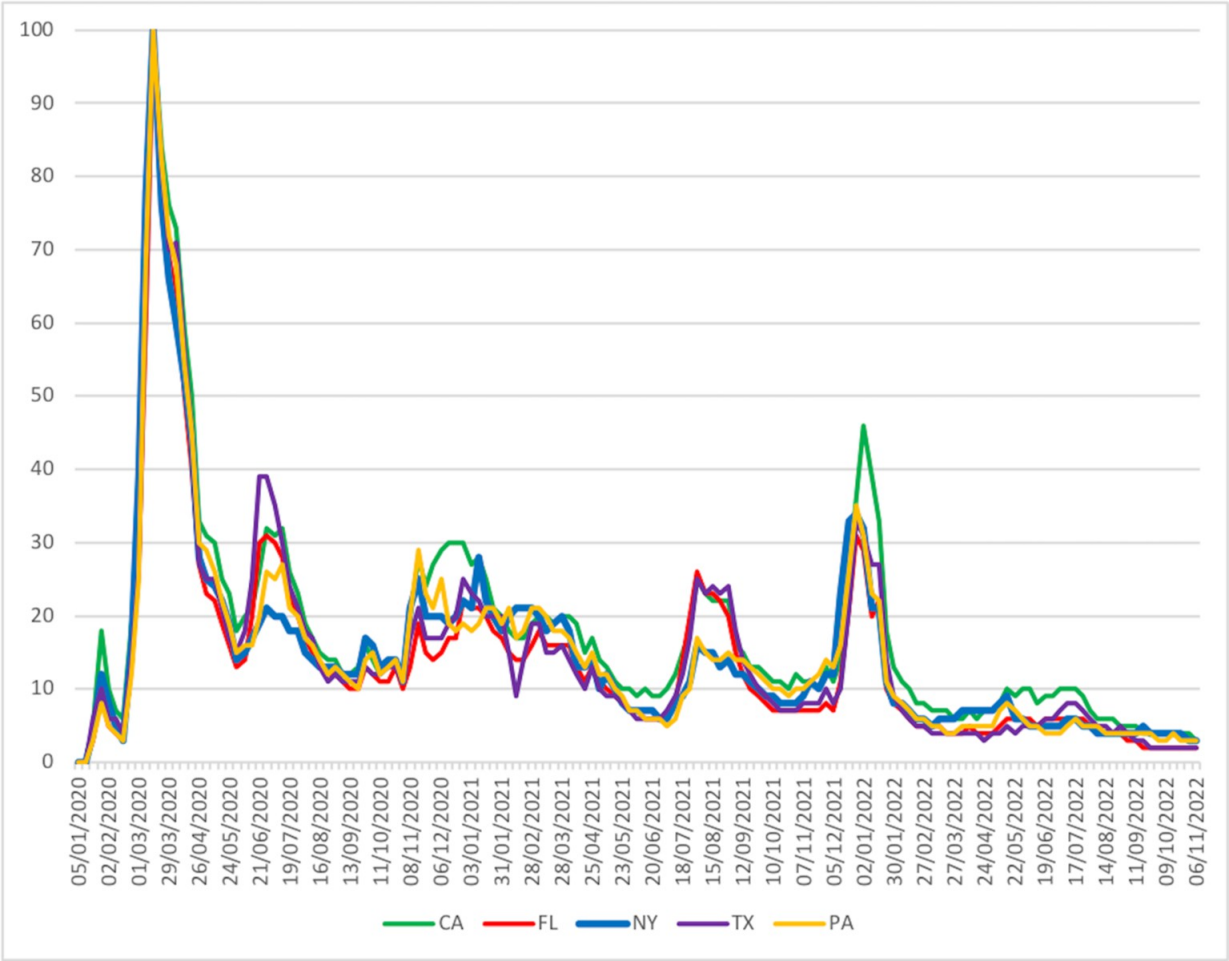
Web-search trends as a measure of public interest for COVID-19. State-level data of the five most populated areas (CA, FL, NY, TX, PA), weekly intervals. Near-perfect concordance of rankings across all 50 states and DC (Kendal’s W 0.94, p<0.001).

Fig 1 illustrates how web search popularity evolved in a sample of the five most populated states. Web search popularity reached its peak in March 2020 to be succeeded by a decline and varying activity around 20% of the peak. On the emergence of Omicron variants by the end of 2021, the relative popularity was temporarily revived without reaching the peak. Notably, high levels were short-lived, and interest fell around 10% of the peak. Also, throughout the observation period, the relative web search popularity never came close to the peak of March 2020. Web-search popularity data are provided as supporting information in S1 Data.

Across the 51 areas (50 states and the District of Columbia), the popularity-mortality trajectories yielded higher similarity indices (median 0.78; interquartile range 0.75–0.82) compared to popularity-incidence trajectories (median 0.74; interquartile range 0.72–0.76, Wilcoxon’s exact p< .001). Noteworthy, the popularity-mortality similarity was very strong (>0.80) in 19/51 (37%) regions, as opposed to the popularity-incidence similarity index, which exceeded 0.80 in only 4/51 (8%) areas (a complete list of estimates is provided in the S1 Appendix).

**Fig 2:**
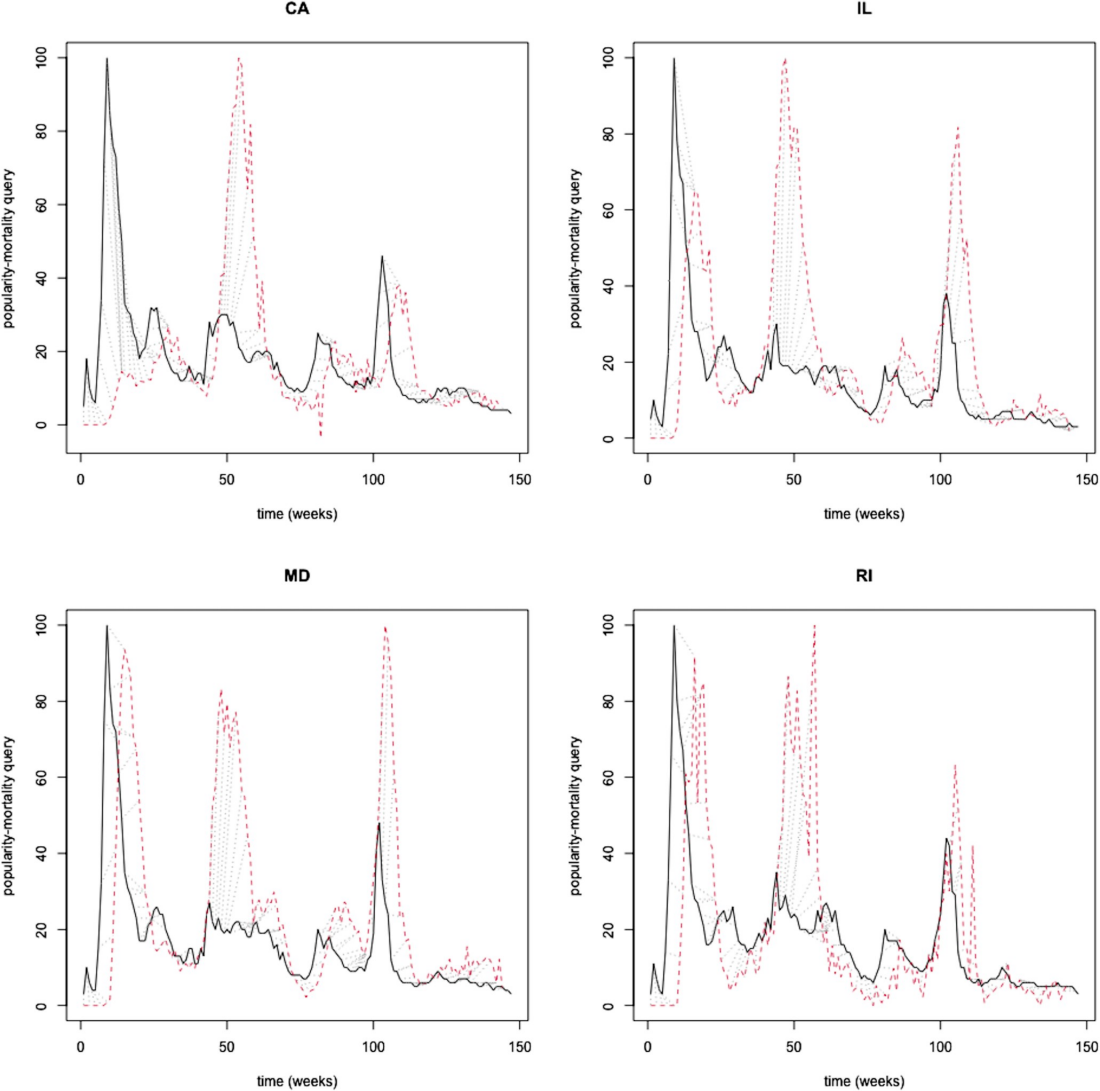
An indicative example of states with a very strong (>0.80) popularity-mortality similarity (CA 0.86, IL 0.86, MD 0.87, RI 0.86). Relative popularity (solid line) and mortality (red line) time series. Volatility in relative popularity preceded changes in COVID-19 mortality that followed. Spikes in web search popularity fell short of the extent of peak mortality during waves.

**Fig 3:**
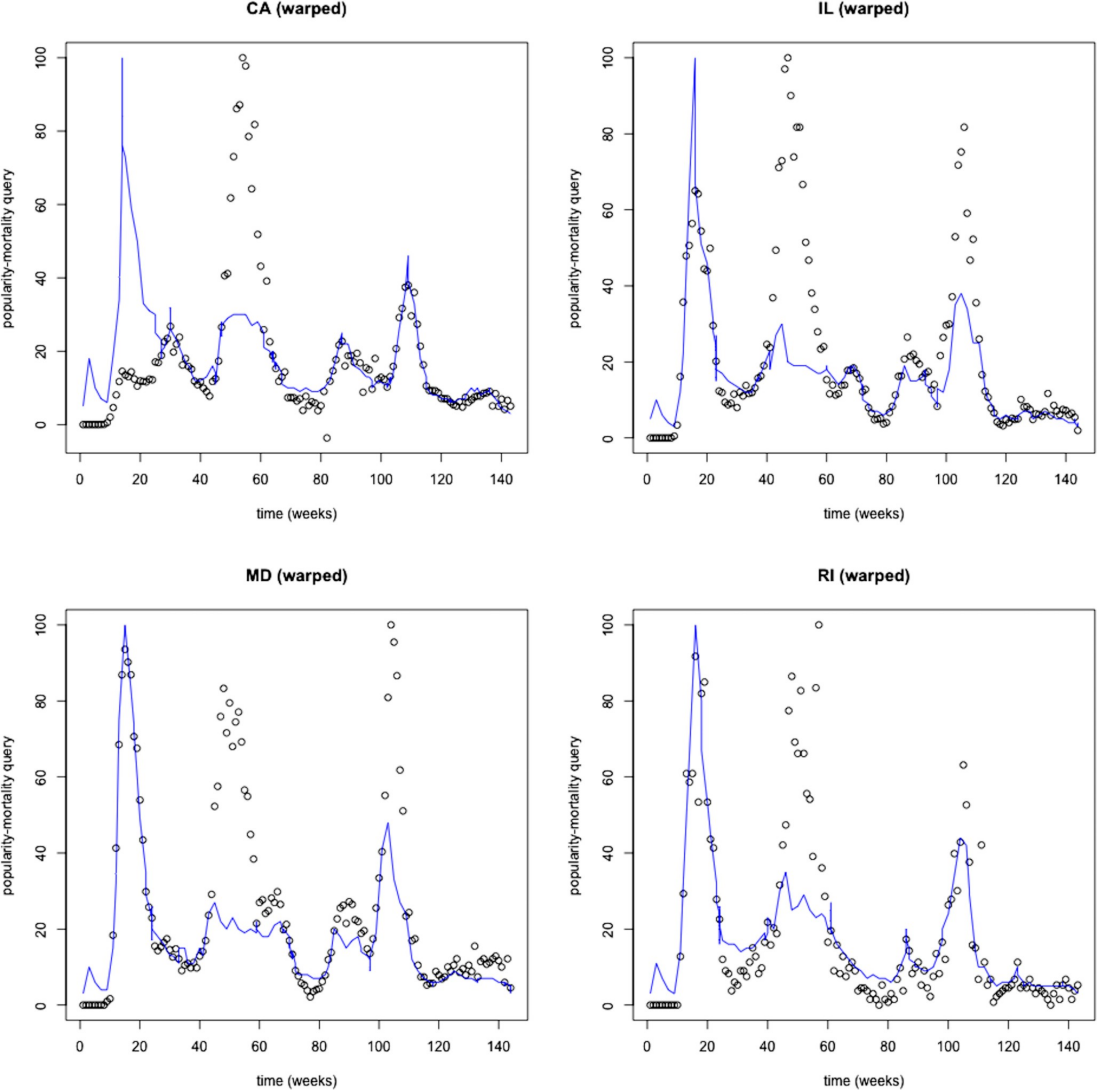
An indicative example of states with a very strong (>0.80) popularity-mortality similarity (CA 0.86, IL 0.86, MD 0.87, RI 0.86) after time-warping for pattern recognition. After alignment, relative popularity (solid line) and mortality (circle markings) as the reference index, show a nearly identical pattern between wave peaks. Popularity underestimated mortality at the peak of waves.

**Fig 4:**
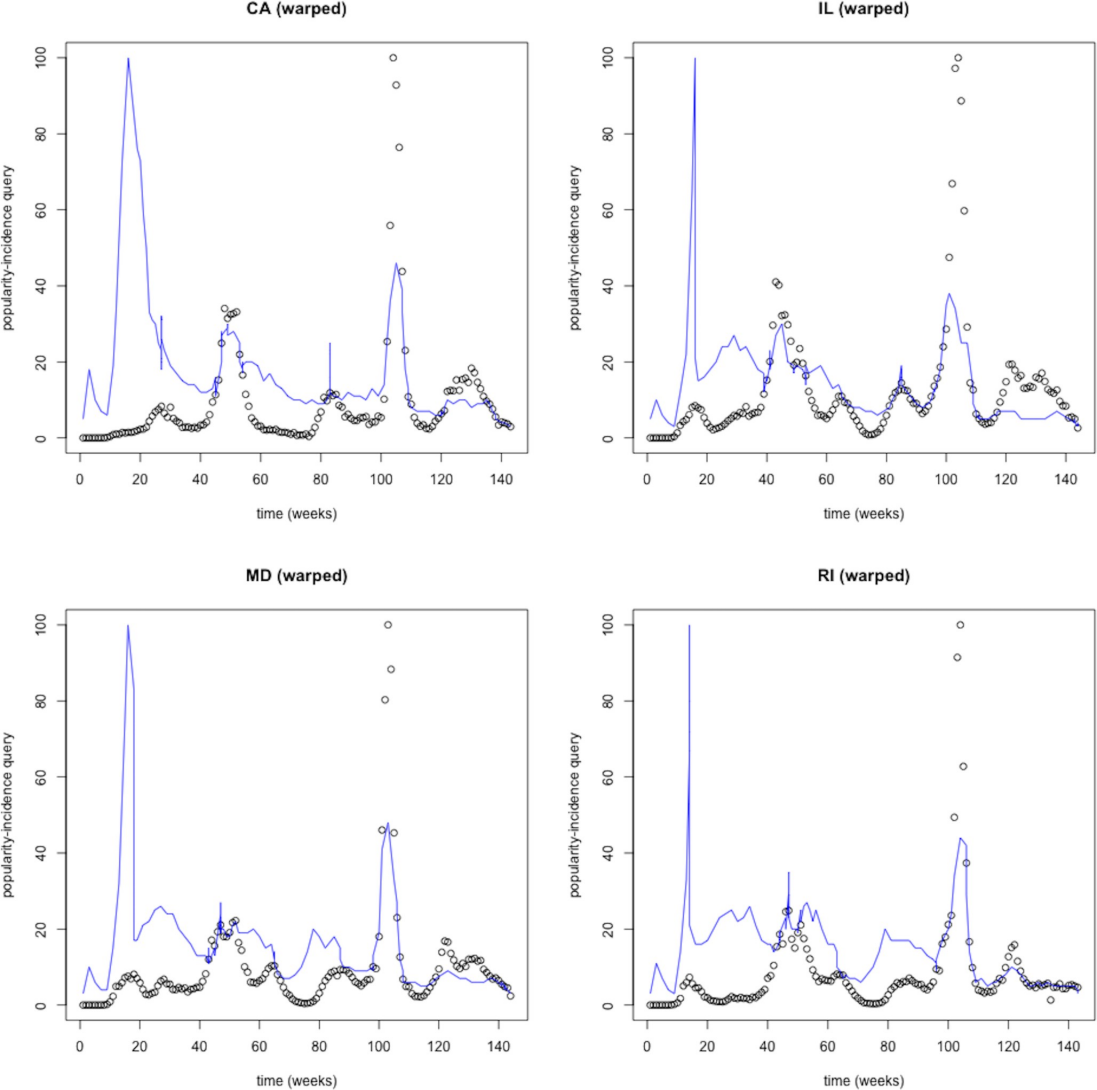
Popularity-incidence similarity for the same four states (CA 0.70, IL 0.76, MD 0.73, RI 0.71) after time-warping for pattern recognition. Relative popularity (solid line) and incidence (circle markings) as the reference index appeared disconnected, particularly between the peaks of COVID-19 waves.

We illustrate an indicative sample of states with a very strong (>0.80) popularity-mortality similarity (CA, IL, MD, RI). On visual assessment, changes in relative popularity preceded the rise in COVID-19 mortality (Fig 2), but spikes did not capture the extent of peak mortality during waves. After alignment, we confirmed that the relative popularity closely matched the mortality trajectory though it underestimated peak mortality during subsequent waves (Fig 3). Instead, popularity-incidence trajectories appeared disconnected, particularly between the peak of waves, during lower COVID-19 activity (Fig 4).

## Discussion

During a pandemic like COVID-19, incidence and related mortality are measures of critical importance for epidemiology and public health [22]. They vary over time to produce a specific longitudinal profile across each geographic division and can be studied retrospectively as a signature of contagion growth. We constructed herein state-level adjusted estimates and used them as a reference index to compare with web-search popularity for COVID-19.

People around the world have reacted with great concern to the emergence of the COVID-19 pandemic, and the web search popularity pattern was impressively similar across states. The web search popularity was high during the initial phase of the pandemic but gradually declined during subsequent waves. The Omicron variant, which appeared in late 2021 and early 2022, revived web search popularity for COVID-19 transiently, to drop to low levels throughout 2022. Historically, societies responded to new threats in an analogous pattern, initially with a great concern or even superstitions, to become detached and indifferent [23,24].

Public interest represents how people perceive the current state of a health emergency and, in this case, the pandemic, while case detection and death counts are objective indicators of contagion growth, with confirmed cases being the most up-to-date and death counts the most lagged. Web searching behaviors are a real-time proxy of public interest [25]. As such, they may capture early the pandemic growth, even before the pertinent indices on incidence and mortality become available. At the state-level analysis, web search popularity for COVID-19 closely matched relative incidence and mortality trajectories, and the popularity of the web searches better resembled the COVID-19 mortality rather than the incidence case trajectory.

Incident cases are grossly miscalculated due to testing and reporting biases, home testing -which is not reported- or no testing at all [26,27]. Furthermore, the public now perceives the virus as less lethal due to previous exposure, vaccinations, and specific treatments [28–32], which may have fueled the decline in interest. People are adapting more efficiently to confront disease, though in parallel, the virus is evolving to be more adaptive [33].

Finally, early 2022 officially signaled a return to normalcy and a shift to preventing the severe consequences of the infection [34]. Consequently, the resulting decline in SARS-CoV-2 testing has led to further underestimating the actual case trajectory [35,36]. On the other hand, death counts are registered three or more weeks after infection, with additional delays in documentation [26,37]. Despite being regarded as a terminal measure, they represent a less biased indicator of the actual state of the pandemic than case detection [26,38], which explains why the relative popularity-mortality trajectories may be a better match than popularity-incidence trajectories. The CDC estimated that only 1 out of 4 COVID-19 infections was reported as opposed to 1 out of 1.32 deaths reported [39], with variation by region, disease severity, and time [40,41].

Public interest is diminished in COVID-19, and it seems unlikely to recover outside the context of a major outbreak or the emergence of a new and deadly escape variant. A progressively deeper understanding of the virus, its transmission ways, and preventive measures mitigate the perceived risk. Prior exposure, mass vaccination, and available treatments provide reassurance to suppress concerns regarding severe outcomes and further diminish public interest [12,42–44]. Societies, in turn, respond with a shift toward normalized behaviors. Half of Americans have returned to their daily routine, reaching a pandemic high, and two-thirds consider COVID-19 a small risk or no risk when returning to their pre-COVID-19 life. Furthermore, although they acknowledge that the fight against COVID-19 should continue, 44% of Americans say that the US has already spent enough and should move on [45].

Our analysis highlighted ex-post that the relative popularity pattern was an impression of the pandemic growth by standard epidemiological measures, namely incidence and mortality. Since web search popularity is a real-time indicator while incidence and mortality are lagged indicators, public interest can be an asset in studying current and future pandemics.

Several studies have explored the association of public interest with aspects of the COVID-19 pandemic, with the majority covering only the initial waves of 2020 [9]. Linear correlation metrics were preferred for data analysis, with a minority of studies (13%) using more complex time-series analysis, including autoregressive integrating moving average and vector-error correction models [9]. Traditional correlation metrics assume a linear relationship and a fixed alignment of events and may not capture time lags and phase shifts. Instead, we used a more flexible methodological approach. Dynamic Time Warping, which is effective for comparing time series data, works well with time lags and non-linear relationships. Furthermore, it outperforms where the emphasis is on the similarity of shapes rather than just the overall correlation [12,19–20].

Limitations of this approach and our analysis are imposed by the survey protocol, the *Google Trends* methodology itself, and a general understanding of the conditions covered [46,47]. In short, there is no standardized protocol for research using *Google Trends*, and research results can vary regarding reproducibility [25]. Additionally, online tool methodologies are regularly changed by providers, and unpublished changes may affect results. Various factors drive public interest, including media coverage, social discourse, and real-world events. Search patterns can be moderated also by engine algorithms, trending topics, or event-related content that is designed to attract attention. While they provide a good real-time proxy of public interest and were widely used during the COVID-19 pandemic [9], giving full credit to web search behaviors simplifies the dynamic nature of public interest. *Google Trends* may reflect public interest in general research topics. Still, complex query organization and multi-term processing are required for qualitative analysis [48,49]. Media influence and public awareness campaigns can moderate web search popularity in the short term through increased interest, social media amplification, and trendsetting [50]. However, their contributions are multifaceted and often qualitative in nature, rendering it an impossible task to extract and measure separately. Furthermore, beyond the initial wave of an emerging disease, where the interest of media and the public rises, it is likely that big media announce and publish only when an increase in incidence and mortality occurs. Therefore, web search trends may better capture these effects as a single composite measure to study for a given period. Finally, universal internet access [51] improves data reproducibility and robustness of estimates, while less developed digital environments and multilingual queries across states may alter associations.

## Conclusion

Web search patterns can mirror public interest, and in this state-level analysis we found that populations react uniformly to the emergence and evolution of a new pandemic. The initial maximal interest in a new threat waned to align with the actual trajectory of the pandemic, particularly the mortality trajectory. Accumulating knowledge, previous exposure, vaccines, and specific treatments attenuated the risk perception and further suppressed interest in COVID-19.

## Supporting information

S1 Appendix State -level estimates. (DOCX)

S1 Data Web-search popularity data across states. (XLSX)
