# Supplementary material for: Public interest trends for COVID-19 and pandemic trajectory: A time-series analysis of US state-level data
Source: PLOS Digit Health. 2024 Mar 12;3(3):e0000462. doi: 10.1371/journal.pdig.0000462 (PMC10932605; doi:10.1371/journal.pdig.0000462)
Supplement: S1 Appendix — (DOCX) [file pdig.0000462.s001.docx]

**S1 Appendix. State -level estimates.** The estimated similarity between the relative web-search popularity for Covid-19 and disease trajectories across 50 states and the District of Columbia. The similarity index is calculated through a dynamic time-warping algorithm.

| state | | Popularity-incidence  similarity | Popularity-mortality  similarity |
| --- | --- | --- | --- |
| AK | Alaska | 0.72 | 0.63 |
| AL | Alabama | 0.79 | 0.78 |
| AR | Arkansas | 0.77 | 0.70 |
| AZ | Arizona | 0.78 | 0.83 |
| CA | California | 0.70 | 0.86 |
| CO | Colorado | 0.74 | 0.84 |
| CT | Connecticut | 0.74 | 0.77 |
| DC | DistrictColumbia | 0.65 | 0.78 |
| DE | Delaware | 0.75 | 0.82 |
| FL | Florida | 0.79 | 0.82 |
| GA | Georgia | 0.81 | 0.73 |
| HI | Hawaii | 0.54 | 0.75 |
| IA | Iowa | 0.76 | 0.81 |
| ID | Idaho | 0.76 | 0.77 |
| IL | Illinois | 0.76 | 0.86 |
| IN | Indiana | 0.76 | 0.85 |
| KS | Kansas | 0.75 | 0.73 |
| KY | Kentucky | 0.74 | 0.78 |
| LA | Louisiana | 0.81 | 0.76 |
| MA | Massachusetts | 0.72 | 0.84 |
| MD | Maryland | 0.73 | 0.87 |
| ME | Maine | 0.69 | 0.75 |
| MI | Michigan | 0.74 | 0.80 |
| MN | Minnesota | 0.72 | 0.80 |
| MO | Missouri | 0.78 | 0.75 |
| MS | Mississippi | 0.81 | 0.81 |
| MT | Montana | 0.71 | 0.75 |
| NC | NorthCarolina | 0.75 | 0.78 |
| ND | NorthDakota | 0.73 | 0.76 |
| NE | Nebraska | 0.77 | 0.78 |
| NH | NewHamshire | 0.72 | 0.84 |
| NJ | NewJersey | 0.72 | 0.82 |
| NM | NewMexico | 0.73 | 0.76 |
| NV | Nevada | 0.81 | 0.78 |
| NY | NewYork | 0.76 | 0.83 |
| OH | Ohio | 0.73 | 0.79 |
| OK | Oklahoma | 0.76 | 0.74 |
| OR | Oregon | 0.72 | 0.68 |
| PA | Pennsylvania | 0.72 | 0.83 |
| RI | RhodeIsland | 0.71 | 0.86 |
| SC | SouthCarolina | 0.77 | 0.80 |
| SD | SouthDakota | 0.74 | 0.75 |
| TN | Tennessee | 0.77 | 0.77 |
| TX | Texas | 0.79 | 0.79 |
| UT | Utah | 0.75 | 0.77 |
| VA | Virginia | 0.75 | 0.82 |
| VT | Vermont | 0.60 | 0.75 |
| WA | Washington | 0.73 | 0.80 |
| WI | Wisconsin | 0.73 | 0.78 |
| WV | WestVirginia | 0.71 | 0.70 |
| WY | Wyoming | 0.72 | 0.70 |
